# Viral RNA detections of enteric pathogens from caprine raw milk in Central Italy

**DOI:** 10.3389/fmicb.2026.1739370

**Published:** 2026-03-16

**Authors:** Gianluigi Ferri, Aurora Astolfi, Filiberto Malatesta, Luca Pennisi, Alberto Vergara

**Affiliations:** 1 Post-Graduate Specialization School in Food Inspection “G. Tiecco”, Department of Veterinary Medicine, University of Teramo, Teramo, Italy; 2 Food and Health Veterinarian Consulting, Teramo, Italy

**Keywords:** food safety, goats, unpasteurized milk, viral foodborne pathogens, zoonoses

## Abstract

**Introduction:**

During primary production, unpasteurized milk may be responsible for the transmission of several foodborne and zoonotic pathogens. Among these, viruses represent a public health concern.

**Methods:**

In this study, 383 raw caprine milk samples were collected from goats farmed at 6 sites (F1–F6) located in the L’Aquila Province (Abruzzo Region, Italy). Molecular assays, including real-time reverse transcription quantitative polymerase chain reaction (RT-qPCR) and nested reverse transcription polymerase chain reaction (RT-PCR), were performed to detect the RNA sequences of astrovirus (AstV), hepatitis A virus (HAV), hepatitis E virus (HEV), norovirus genogroups I and II (NoV-GI and NoV-GII), and rotavirus (RV).

**Results:**

Results showed that 15.40% (59/383) of samples were positive for at least one pathogen, and 3.39% (13/383) co-detected AstV, HEV, and/or NoV-GI, with the highest frequency in samples collected from goats farmed at F5 and F6. Among the viruses investigated, HEV RNA was detected in samples from all farms, representing 7.57% (29/383), followed by AstV at 6.00% (23/383) and NoV-GI at 5.74% (22/383).

**Discussion:**

Although the majority of the viruses have been detected in faecal samples in previous studies, this study provides original data on their presence in unpasteurized caprine milk in Italy.

## 1 Introduction

Primary production systems have been identified as the majority of probable sources of foodborne pathogens caused by viruses and bacteria, which are considered in many risk assessments and risk-based analyses in different food production chains involving foods of animal origin (Pakbin et al., 2022; Santos-Silva et al., 2022). Depending on the viral genotypes or genogroups involved, clinically appreciable symptoms may occur; they may cause non-specific gastrointestinal symptoms, and the severity differs depending on the immunocompetence profiles of the infected hosts. Based on the different epidemiological distributions of the involved viral strains, these conditions characterize and influence specific areas located in both developed and developing countries (El-Senousy et al., 2020).

Viral ultrastructural characteristics, such as non-enveloped or quasi-enveloped viruses, confer long-term persistence in many different environments (both terrestrial and marine); for these reasons, they are also defined as emerging and re-emerging viruses (Purdy et al., 2017). Among both domestic and wild animal species, many serve as reservoirs or spillover hosts (such as wild boars and wild ruminants) for viral microorganisms. Furthermore, the environmental sharing of grazing areas in extensive and semi-extensive farming systems provides favorable conditions for possible cross-species infections (Karesh et al., 2012). These animals, usually used as food for human consumption, can harbor zoonotic viral foodborne pathogens transmitted to consumers, particularly through foods that are ingested raw or undercooked (Ozogul et al., 2025). Among the primary production systems, unpasteurized milk and derived processed products have been considered potential routes of viral and bacterial transmission because of the absence of thermal treatment (Fusco et al., 2020). Focusing on the viral pathogens, the majority of reported RNA viruses from raw milk samples, detected in mammals and humans (Rivero-Juarez et al., 2016), are enteric ones, including astrovirus (AstV), hepatitis A virus (HAV), hepatitis E virus (HEV), norovirus genogroups I and II (NoV-GI and NoV-GII), and rotavirus (RV) (Demirci et al., 2019). In recent decades, these viruses (AstV, HAV, HEV, NoV-GI, NoV-GII, and RV) have attracted increasing attention, particularly in food-producing animal species (detailed information is provided in Table 1).

**Table 1:** Taxonomic information about the considered foodborne pathogens.

Virus	Family	Genus	Z/NZ	References
AstV	Astroviridae	*Astrovirus*	NZ-AH	Bosch et al. (2019)
HAV	Picornaviridae	*Hepatovirus*	H	Zell et al. (2017)
HEV	Hepeviridae	*Paslahepevirus*	Z-H-AH	Purdy et al. (2022)
NoV-GI and GII	Caliciviridae	*Norovirus*	H-AH	Vinjé et al. (2019)
RV	Sedoreoviridae	*Rotavirus*	H-AH	Matthijnssens et al. (2022)

AH, animal host; H, human host; Z, zoonotic viruses; NZ, not zoonotic viruses.

However, few studies have investigated small dairy ruminant species compared with cattle (Dziedzinska et al., 2020). More recently, HEV RNA, among foodborne viral pathogens, has been mainly investigated and detected in ovine unpasteurized milk obtained from animals farmed in Europe (Demirci et al., 2019; Dziedzinska et al., 2020; Ferri et al., 2024). In contrast, the caprine species has been marginally screened, as observed in two recent studies, Demirci et al. (2019) and Dziedzinska et al. (2020). In parallel, the epidemiological studies have expanded their scientific designs and approaches through the involvement of multiple viral detections. Indeed, Pakbin et al. (2022) were the first to detect different viral RNA sequences, belonging to zoonotic pathogens (e.g., AstV, HAV, NoV-GI, bovine leukemia virus, and tick-borne encephalitis virus), in raw milk samples collected from cows farmed in Iran. For this purpose, this study, from a comparative perspective, aimed to screen raw milk samples collected from 383 goats (considered possible reservoir and/or spillover hosts) farmed at six sites distributed in L’Aquila Province (Abruzzo Region, Italy). The animals were managed under a semi-extensive farming system with access to seasonal grazing during spring and summer seasons and in closed sheds during the cold months. Molecular methods such as RT-qPCR and nested RT-PCR were performed to detect AstV, HAV, HEV, NoV-GI, NoV-GII, and RV RNA. Therefore, this investigation aims to provide original data on the *silent* circulation of viral RNA from foodborne pathogens and their epidemiological implications on food safety.

## 2 Materials and methods

### 2.1 Samples collection

**Figure 1:**
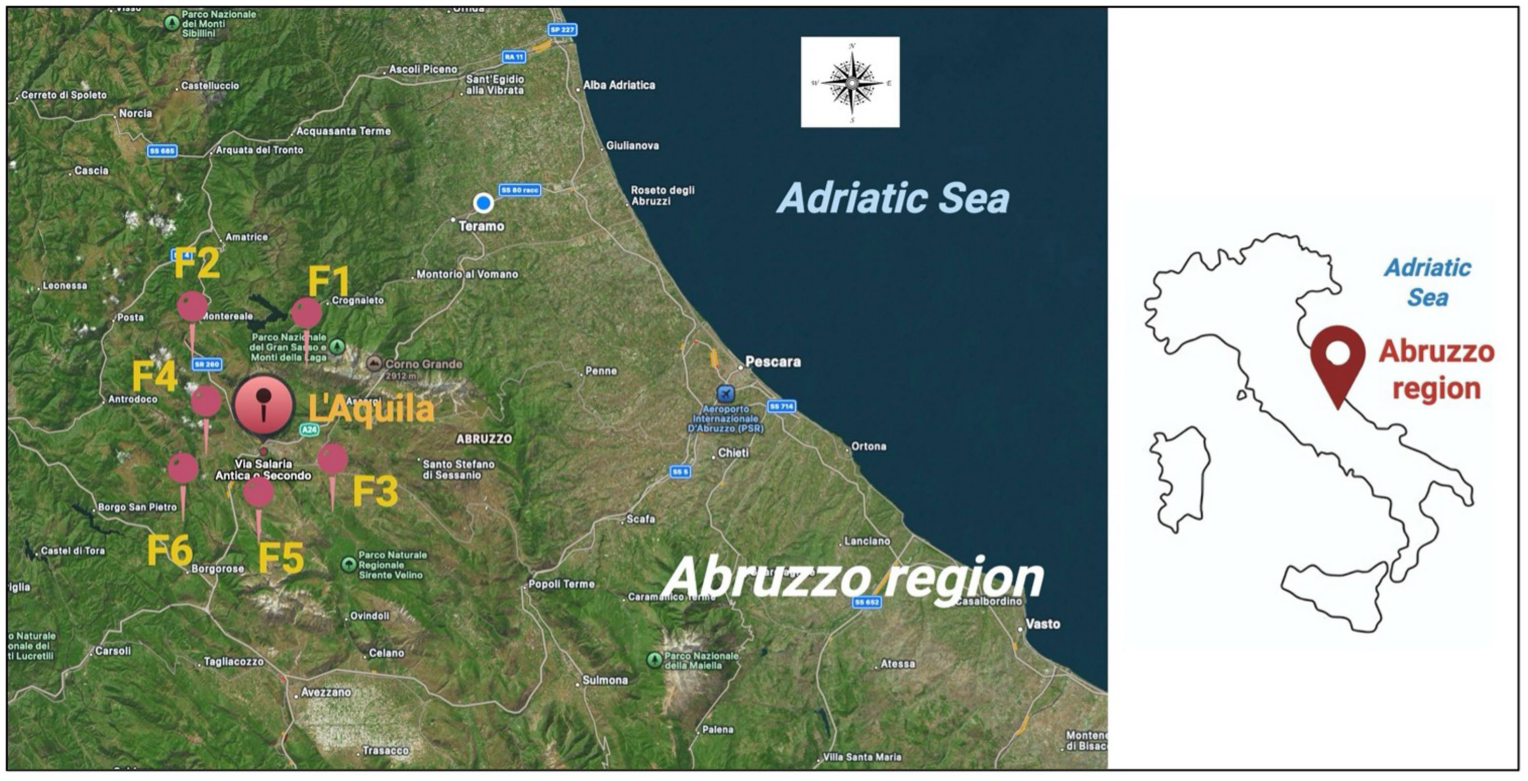
Geographical distribution of the screened farms in L’Aquila Province (Abruzzo Region, Italy) involved in this study.

Between March 2024 and April 2025, 383 primiparous and pluriparous female goats (*Capra aegagrus hircus*) were included in this study. The screened animals presented an average age of 2 ± 1.5 years and a weight of 45 ± 10 kg, farmed at six small and rural semi-extensive farms (F1–F6) located in the L’Aquila Province, Abruzzo Region (Italy). These sites are located within two National Parks: Gran Sasso National Park (surface: 1,413 km^2^) and Sirente Velino National Park (surface: 543.6 km^2^), normally populated by wild animal species such as wild boars (*Sus scrofa*) and wild ruminants (*Rupicapra pyrenaica ornata*), presenting an average population density of 3.8/100 ha (Regional Abruzzo Hunting Report and ISPRA, 2023). Based on the applied semi-extensive farming systems, in the spring and summer seasons, breeders usually feed animals on seasonal grazing areas, which goats possibly share with wild animal species (Figure 1).

Aliquots (50 mL per animal) of unpasteurized caprine milk were collected individually, during the lactation peaks, by farmers after performing the pre- and post-dipping procedures during peak lactation by using udder cleaning followed by chloride-based sanitation. After collection, samples were transported to the laboratory under refrigerated conditions. A detailed distribution of the screened animals per farm is illustrated in Table 2.

**Table 2:** Screened milk/animal samples collected in six caprine farms located in the Abruzzo Region (Italy).

Screened goats	Farms	Screened animals
383 goats (*Capra aegagrus hircus*)	F1	38
	F2	105
	F3	75
	F4	61
	F5	54
	F6	50

F, farm.

The food matrices were studied for the detection of RNA sequences belonging to six different zoonotic viral foodborne pathogens. AstV, HAV, HEV, NoV-GI, NoV-GII, and RV were considered based on two different milk contamination pathways: (1) the physiopathological one occurs when virions are excreted by infected animals during the viremia through the mammary gland, and (2) fecal contamination of mammary glands is based on the improper sanitation procedures and handling, demonstrating possible biological hazards for consumers if the milk is consumed raw, as observed by Pakbin et al. (2022).

### 2.2 RNA extraction and efficacy evaluation

The first step was the collection of five aliquots of 2.5 mL of caprine (*Capra aegagrus hircus*) raw milk obtained from the initial collected volume of 50 mL per animal, and the efficacy evaluation of the performed assays was allowed by spiking, adding 5 × 10^6^ MS2-phage-like particles (MS2-PLP), in accordance with methods described by Mikel et al. (2016). The next step required pooling the five aliquots (2.5 mL) coming from the same animal (action was required for the previous step) into a single volume of 12.5 mL to allow physical separation from the natural fat layer. These samples were centrifuged at 3,000 ×g for 15 min under refrigerated conditions. This step was followed by fat layer removal, and successively, 200 μL of HCl solution 1 M (pH 3.5–4.0) was added to all tubes containing centrifuged milk samples in order to induce proteins and viral genomic fragment precipitation. This method, described by Dziedzinska et al. (2020), included a second centrifugation under the following conditions: 4,000 × g for 10 min (at 4 °C). The obtained supernatant was removed, and pellets were resuspended using 5 mL of sterile PBS (pH 7.2), which was used in the viral RNA extraction procedures using the TRIzol LS method (Invitrogen Ltd., Paisley, UK). Specifically, 250 μL of TRIzol reagent and 100 μL of chloroform were added to 500 μL of RNA template per animal. After centrifugation (12,000 × g for 15 min), specimens were washed twice with 500 μL of isopropyl and ethyl alcohols (75% concentration), respectively. A final centrifugation at 7,500 × g for 5 min was performed, and the obtained RNA pellets were resuspended with 50 μL of sterile RNase-free water (Invitrogen UltraPure DNase/RNase-Free Distilled Water, ThermoFisher™, Waltham, MA, United States). All samples were stored at −80 °C until molecular analysis. For all positive pools, each milk aliquot per animal was screened following the above-mentioned procedures to identify the specific positive samples.

### 2.3 Enteric viral RNA detections: real-time RT-qPCR and nested RT-PCR assays

Quantitative assays (real-time RT-qPCR) were performed to amplify specific RNA genomic regions (expressed as GE/mL) belonging to AstV, HAV, HEV, NoV-GI, NoV-GII, and RV by using specific commercial kits named KQASV, KQHAV, KQHEV, KQNVGI, KQNVG, and KQRV (ceeramTools—Thermo Fisher Scientific™, Waltham, MA, United States), respectively. The quantitative data obtained, expressed as GE/mL, produced curves that were compared with standard curves at known concentrations: from 10^7^ GE/mL to 10^1^ GE/mL. In accordance with manufacturer instructions, all real-time reactions were performed in 25 μL (total volume), and positive and negative controls were included in all reactions (provided by the used commercial kits). The starting sterile mix volume was 20 μL, and 5 μL of extracted RNA was added to each reaction. The fluorescence channel was set at 520, and the established limit of detection (LOD) and limit of quantification (LOQ) referred to a 10-fold dilution (from 10^7^ GE/mL to 10^1^ GE/mL). The LOD parameter was represented by the lowest MS2-PLP concentration, and the smallest amount of the screened analyte was described as the LOQ value with a respective coefficient of variation 25% (Kralik and Ricchi, 2017). For all considered viral pathogens, the same thermocycler settings were used: initial reverse transcription (1) 45 °C for 10 min, denaturation (2) 95 °C for 10 min, and 40 cycles (3) including denaturation (3a) 95 °C for 15 s, annealing (3b) 60 °C for 45 s, and extension (3c) 72 °C for 45 s, as illustrated by manufacturer instructions (CeramTools—Thermo Fisher Scientific™, Waltham, MA, United States).

Quantitative assays were followed by qualitative RT-PCR and nested RT-PCR. Table 3 shows the screened viral pathogens and the respective primers. All reaction volumes and thermocycler settings agreed with references.

**Table 3:** Viral pathogens, targeting genes, and primers used for RT-PCR and nested RT-PCR assays in this study.

Viruses	Genes	Primers	Sequences (5′-3′)	Amplicon (bp)	References
AstV	ORF2	Mon245 (Fw)	TTAGTGAGCCACCAGCCATC	413 bp	Noel et al. (1995)
		Mon244 (Rev)	GGTGTCACAGGACCAAAACC		
		Mon270 (Fw)	TCAGATGCATTGTCATTGGT	449 bp	
		Mon269 (Rev)	CAACTCAGGAAACAGGGTGT		
HAV	VP1/2A junction	+2,897 (Fw)	TATTCAGATTGCAAATTAYAA	420 bp	Taffon et al. (2011)
		−3,288 (Rev)	AAYTTCATYATTTTCATGCTCCT		
		+2,949 (Fw)	TATTTGTCTGTYACAGAACAATCAG	244 bp	
		−3,192 (Rev)	AGGRGGTGGAAGYACTTCATTTG		
HEV	ORF1	HEV-cs (Fw)	TCGCGCATCACMTTYTTCCARAA	470 bp	Johne et al. (2010)
		HEV-cas (Rev)	GCCATGTTCCAGACDGTRTTCCA		
		HEV-csn (Fw)	TGTGCTCTGTTGGCCCNTGGTTYG	333 bp	
		HEV-casn (Rev)	CCAGGCTCACCRGARTGYTTCTTCCA		
	ORF2	3,156 (Fw)	AATTATGCCTCAGTACTCGGAGTTG	731 bp	Huang et al. (2002)
		3,157 (Rev)	CCCTTAGTCCTTGCTGACGCATTCTC		
		3,158 (Fw)	GTTAATGCTTCTGCATATCATGGCT	348 bp	
		3,159 (Rev)	AGCCGACGAAATCAATTCTGTC		
NoV-GI & NoV-GII	RdRp region	MR3 (Fw)	CCGTCAGAGTGGGTATGAA	470 bp	Lew et al. (1994)
		MR4 (Rev)	AGTGGGTTTGAGGCCGTA		
		Yuri22 (Fw)	ATGAATGAGGATGGACCCAT	370 bp	
		Yuri22 (Rev)	CATCATCCCCGTAGAAAGAT		
RV	PRV-A	P1 (Fw)	GGCTTTTAAAGCGCTACAGTGATGTCTCT	317 bp	Ben Salem et al. (2010)
		P2 (Rev)	GGTCGTGATTGTGTTGATGAATCCATAGA		
		P3 (Fw)	CTCAGCATTGACGTAACGAGTCTTCC	208 bp	
		P4 (Rev)	TGAGTGGATCGTTTGAAGCAGAATCAGA		

ORF, Overlapping open reading frames; rt, reverse transcription; nes, nested PCR; D = A, G, T; M = A or C; N = A, C, G, T; R = A or G; Y = C or T; bp: base pairs.

**Figure 2:**
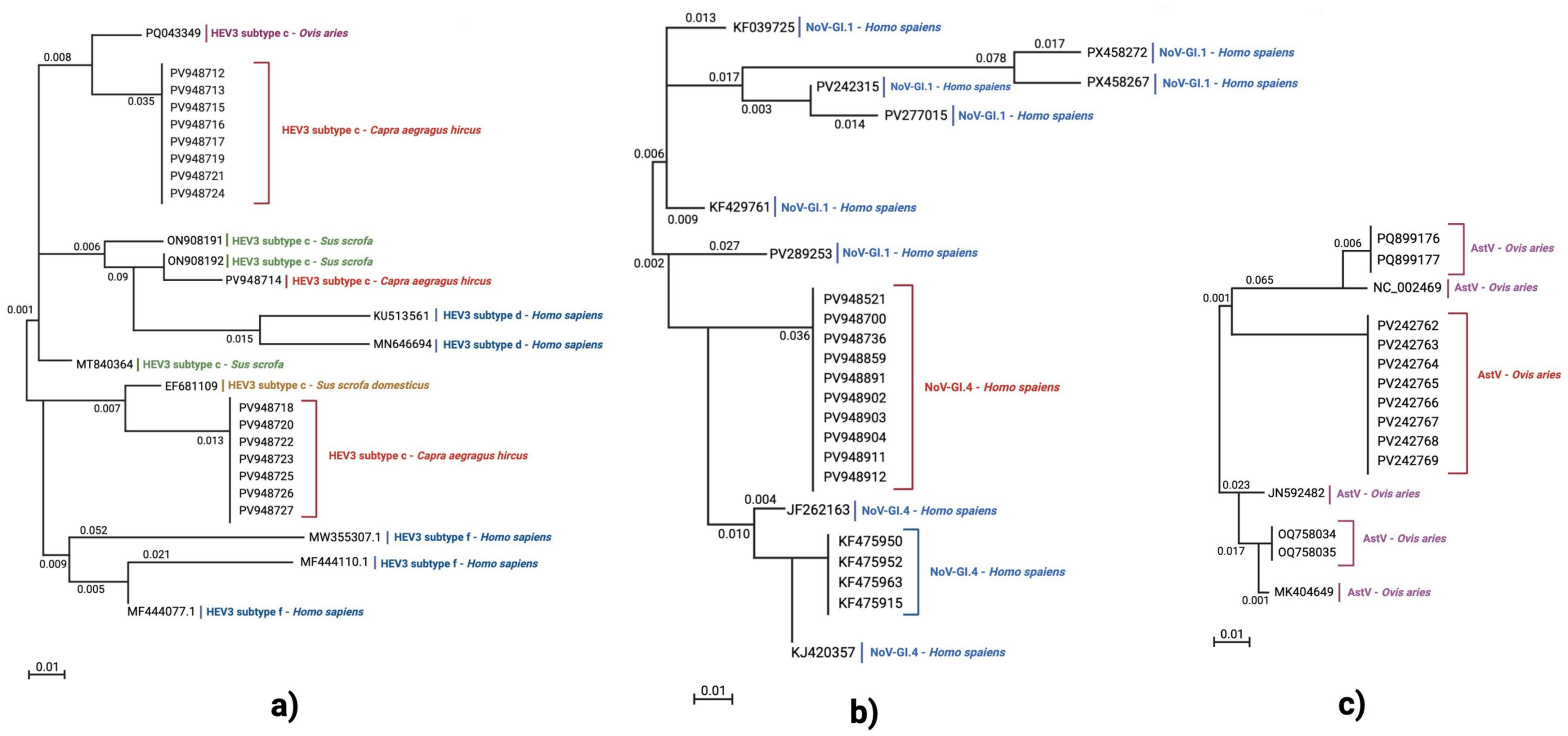
Neighbor-joining phylogenetic trees were constructed based on the detected amplicons belonging to the following: **(a)** HEV, **(b)** NoV-GI, and **(c)** AstV. Neighbor-joining phylogenetic trees were constructed using the *p*-distance model with bootstrapping of 1,000 replicates (0.01 distance scale). All sequences identified in this study are highlighted in red. These trees were constructed based on fragments belonging to HEV, NoV-GI, and AstV as follows: **(a)** HEV tree based on a 348 bp fragment of the ORF2 gene; **(b)** NoV-GI related to a 370 bp fragment of the *RdRp* gene region; **(c)** AstV based on a 449 bp fragment of the ORF2 gene.

All RT-PCR and nested RT-PCR products were evaluated by electrophoresis at different concentrations of agarose gel (1.5–2.0% based on the amplicon size), and the respective running set was 85 V for 40 min. Results interpretation was performed by comparing amplicons to specific commercial DNA ladders (50 bp, 100 bp, 150 bp) (Genetics, FastGene®, Düren, Germany). The expected amplicons were confirmed after purification (using the Qiagen QIAquick® PCR Purification Kit, Hilden, Germany) and Sanger sequencing performed by Biofab Laboratories (Rome, Italy). The nucleotide similarity and alignment tests were performed for all suspected positive amplicons using the BLASTN system.^1^ Finally, they were loaded into the GenBank platform^2^ and released with their own accession numbers (accessed on 17, 18, and 20 July 2025). The published sequences were also involved in the evolutionary analyses that were performed by using the MEGA X software, as described by Kumar et al. (2018). Phylogenetic trees were constructed following the Neighbor-joining method (Saitou and Nei, 1987), as represented in Figure 2 in the Results section.

### 2.4 Statistical analysis

Statistical analyses were designed in two steps. The first was descriptive, and the normality assumption was evaluated by performing the Shapiro–Wilk test, considering the alpha value significant at <0.05. The two-tailed *t*-test was used to compare the detected viral GE/mL (dependent variables) across farm distributions. Finally, the 95% confidence intervals (CI) were calculated for all percentages when applicable.

## 3 Results

The 383 unpasteurized milk samples collected from goats (*Capra aegagrus hircus*) were analyzed in order to detect RNA fragments of the investigated pathogens. Overall, 15.40% (59/383; 95% CI: 11.79–19.01%) of the screened milk samples were positive for at least one RNA sequence from the five screened viral pathogens. The overall positivity was 19.06% (73/383; 95% CI: 15.13–22.99%) when co-detection profiles were considered. The observed prevalences, per individual detected pathogen, were as follows: 6.00% (23/383; 95% CI: 3.62–8.38%) AstV, 7.57% (29/383; 95% CI: 4.92–10.22%) HEV, and 5.74% (22/383; 95% CI: 3.41–8.07%) NoV-GI. HAV, NoV-GII, and RV RNA were not detected in milk specimens from the six screened farms.

Among the positive samples, AstV RNA (ORF-2 gene) was detected in 38.98% (23/59; 95% CI: 26.54–51.42%), of which 16.94% were exclusively positive for AstV; 49.15% (29/59; 95% CI: 36.39–61.91%) were positive for HEV RNA, of which 33.89% (20/59; 95% CI: 21.81–45.97%) were positive for single detection. NoV-GI was detected in 37.29% (22/59; 95% CI: 24.95–49.63%), of which 27.11% (16/59; 95% CI: 16.95–37.27%) represented single-pathogen detection.

Based on the detected pathogens, HEV RNA was the most frequently detected virus; it was found in samples collected from all six screened farms (F1–F6). The obtained prevalences [combined positivity: 49.15% (29/59; 95% CI: 36.39–61.91%) and as exclusive detections: 33.89% (20/59; 95% CI: 21.81–45.97%)] demonstrated the wide distribution of HEV RNA among caprine farms in L’Aquila Province.

Concerning viral detections and distributions per farm, the observed prevalence values of positive samples for all considered viruses were: 5.26% (2/38; 95% CI: 0.17–10.35%) in F1, 6.67% (7/105; 95% CI: 0.19–11.44%) in F2, 10.67% (8/75; 95% CI: 3.69–17.65%) in F3, 6.56% (4/61; 95% CI: 0.35–12.77%) in F4, 42.59% (23/54; 95% CI: 29.41–55.77%) in F5, and 28.00% (14/50; 95% CI: 15.56–40.44%) in F6.

Considering RNA positivity per farm, the following prevalences were observed: 3.39% (2/59; 95% CI: 0.27–6.51%) in F1, 11.86% (7/59; 95% CI: 3.61–20.11%) in F2, 13.56% (8/59; 95% CI: 4.82–22.30%) in F3, 6.78% (4/59; 95% CI: 0.37–13.19%) in F4, 38.98% (23/59; 95% CI: 26.54–51.42%) in F5, and 25.42% (15/59; 95% CI: 14.31–36.53%) in F6.

In both mentioned perspectives, F5 revealed the highest prevalence values when compared with the others, followed by F6. Indeed, the performed *t*-test also permitted highlighting statistically significant differences (*p-*value < 0.001) between F5 and all other farms (F1, F2, F3, F4, and F6), considering positive sample distributions across farms as independent variables.

Focusing on screened pathogens per farm, HEV was detected in F1 to F6 (as previously described) among the screened caprine population. The following prevalences were observed: 5.26% (2/38; 95% CI: 0.17–10.35%) in F1, 2.85% (3/105; 95% CI: 0.06–5.64%) in F2, 5.33% (4/75; 95% CI: 0.25–10.41%) in F3, 6.56% (4/61; 95% CI: 0.35–12.77%) in F4, 16.67% (9/54; 95% CI: 6.73–26.61%) in F5, and 14.00% (7/50; 95% CI: 5.97–22.03%) in F6.

AstV RNA sequences were not detected from F1, F3, and F4; however, they were detected in the others: 1.90% (2/105; 95% CI: 0.07–3.73%) in F2, 22.22% (12/54; 95% CI: 11.14–33.30%) in F5, and 18.00% (9/50; 95% CI: 7.35–28.65%) in F6.

Finally, NoV-GI, except for F1 and F4, was detected in F2 at 2.86% (3/105; 95% CI: 0.32–6.04%) in F2, 5.33% (4/75; 95% CI: 0.25–10.41%) in F3, 18.52% (10/54; 95% CI: 8.16–28.88%) in F5, and 8.00% (4/50; 95% CI: 0.48–15.52%) in F6. The statistical analysis allowed for highlighting statistically significant differences between F5 and F2, F3, and F6 with *p*-values of <0.001.

Among the tested samples, 3.39% (13/383; 95% CI: 1.58–5.20%) showed co-detection of two (2-CoH) or three (3-CoH) viral RNA sequences. A different prevalence value of 22.03% (13/59; 95% CI: 11.46–32.60%) could be observed if only positive ones were considered. RNA sequences were detected in the 84.61% (11/13; 95% CI: 65.00–100.00%) of the samples: AstV-HEV 63.63% (7/11; 95% CI: 35.20–92.06%) and AstV-NoV-GI 36.36% (4/11; 95% CI: 7.93–64.79%) (Figure 2). Finally, 15.38% (2/13; 95% CI: 0.02–30.74%) simultaneously showed RNA sequences belonging to AstV, HEV, and NoV-GI. In all 2-CoH or 3-CoH-positive milk samples, AstV RNA sequences were detected.

Based on the different distributions of positive multiple harboring specimens per farm, 53.85% (7/13; 95% CI: 26.76–80.94%) of them were collected from F5, 38.46% (5/13; 95% CI: 12.01–64.91%) from F6, and 7.69% (1/13; 95% CI: 0.26–15.12%) from F2. F1, F3, and F4 did not show 2-CoH and/or 3-CoH profiles.

Focusing on the detected viral RNA amounts (GE/mL) per farm, the highest values (10^2^ GE/mL) were observed only in F5, which showed statistically significant differences compared with F6 (F5-F6; *p* = 0.001) and F2 (*p*-value < 0.001).

The quantitative data (GE/mL), obtained by performing the RT-qPCR assays, showed an average amount of 10^2^ GE/mL. Positive samples, collected from F5 and F6, showed the highest HEV RNA loads (10^2^ GE/mL) compared to the other farms, such as F1 and F2, which showed the lowest values (10^1^ GE/mL) (Figure 2). The performed *t*-test showed statistically significant differences: F5-F1 and F5-F2 showed final *p*-values of <0.001, whereas F6-F1 and F6-F2 showed *p* = 0.003.

The highest AstV RNA loads (10^2^ GE/mL) were observed in F5 (26.08% or 6/23; 95% CI: 8.14–44.02%), followed by F6 (8.69% or 2/23; 95% CI: 0.36–17.02%). Furthermore, the two-tailed *t*-test showed a statistically significant difference considering the detected viral loads (GE/mL) and their respective collecting farms: F5-F6 (*p* < 0.001).

Finally, NoV-GI presented an average value of 10^1^ GE/mL NoV-GI RNA.

**Figure 3:**
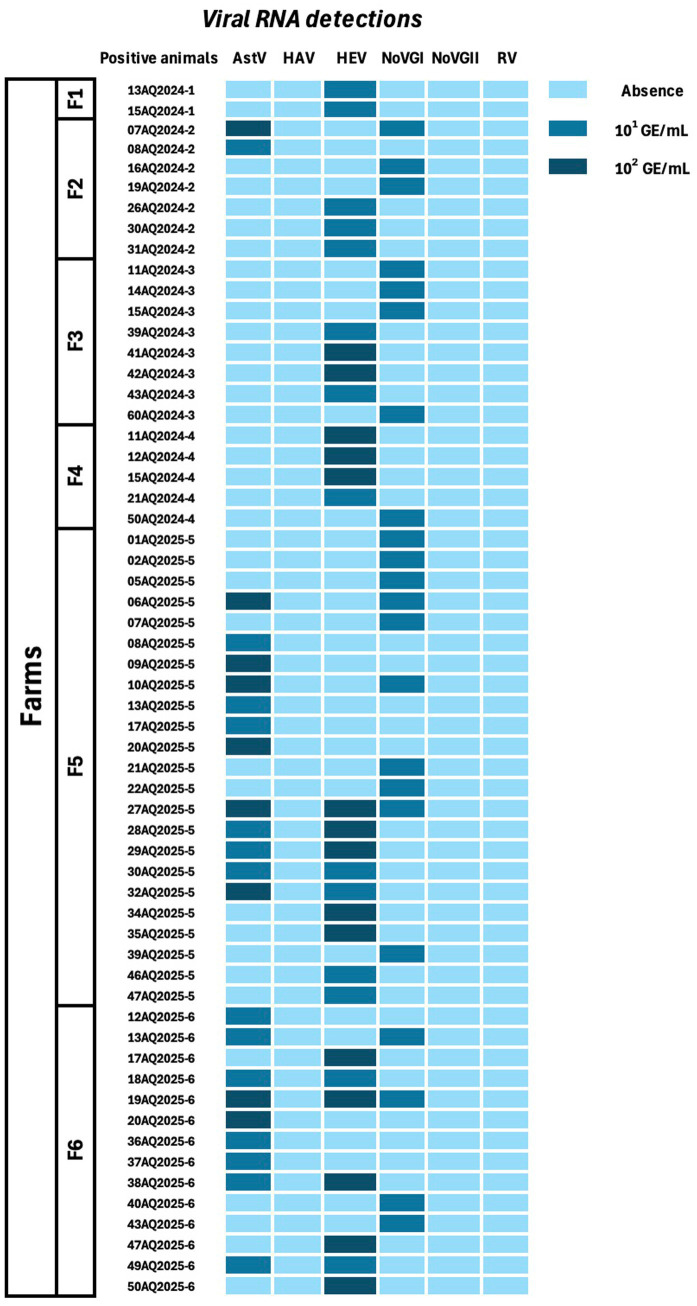
Heatmap of positive animals for the screened viral RNA genomic fragments (GE/mL) amplified from the raw milk samples.

For all considered pathogens, the electrophoresis assays were performed for the nested RT-PCR products, and the expected amplicons were sequenced (as described in the Materials and Methods section). A schematic representation of a positive milk sample-subject-farm-viral RNA as a heatmap is in Figure 3.

Starting from HEV, the amplification products (ORF-2 gene) of nested RT-PCR were sequenced, as previously described in the Method section, and analyzed using the BLASTN tool for nucleotide alignments, showing high similarity (100.0%) to the HEV genotype 3 subtype c. From comparisons among the obtained sequences, nucleotide identities ranged between 95.0 and 98.0%.

All the HEV confirmed sequences were deposited and registered in the GenBank platform under the following accession numbers: PV948712, PV948713, PV948714, PV948715, PV948716, PV948717, PV948718, PV948719, PV948720, PV948721, PV948722, PV948723, PV948724, PV948725, PV948726, and PV948727 (Figure 2). A schematic representation of RNA sequences per pathogen and their respective registration data is presented in Table 4.

**Table 4:** Detected viral RNA sequences per pathogen and the respective registered GenBank accession numbers: a schematic representation.

Pathogens	Target genes	Farms	Localization	Collection date	GenBank a.n.	Sequences length (bp)
HEV	ORF2	F1	Onna (AQ) 42.19 N; 13.28E	25 March 2025	PV948712	276 bp
		F2			PV948713	276 bp
					PV948714	299 bp
		F3	Roio Piana (AQ) 42.19 N;13.21E	05 May 2025	PV948715	286 bp
					PV948716	276 bp
		F4	Bagno Grande (AQ) 42.18 N; 13.25E	12 May 2025	PV948717	286 bp
					PV948718	398 bp
		F5		24 April 2025	PV948719	286 bp
					PV948720	398 bp
					PV948721	276 bp
					PV948722	398 bp
					PV948723	398 bp
		F6	Poggio Roio (AQ) 42.19 N; 13.22 E	21 May 2025	PV948724	251 bp
					PV948725	398 bp
					PV948726	276 bp
					PV948727	276 bp
AstV	ORF2	F2	Onna (AQ) 42.19 N; 13.28E	25 March 2025	PV972762	460 bp
		F5	Bagno Grande (AQ) 42.18 N; 13.25E	24 April 2025	PV972763	257 bp
					PV972764	400 bp
					PV972765	360 bp
					PV972766	460 bp
		F6	Poggio Roio (AQ) 42.19 N; 13.22 E	21 May 2025	PV972767	360 bp
					PV972768	256 bp
					PV972769	397 bp
NoV-GI	Rdrp	F2	Onna (AQ) 42.19 N; 13.28E	25 March 2025	PV948521	563 bp
		F3	Roio Piana (AQ) 42.19 N;13.21E	05 May 2025	PV948700	480 bp
					PV948736	415 bp
		F5	Bagno Grande (AQ) 42.18 N; 13.25E	24 April 2025	PV948859	559 bp
					PV948891	480 bp
					PV948902	244 bp
					PV948903	563 bp
					PV948904	297 bp
		F6	Poggio Roio (AQ) 42.19 N; 13.22 E	21 May 2025	PV948911	363 bp
					PV948912	368 bp

GenBank a.n., GenBank accession number; F, farms.

Among the HEV ORF2 registered sequences presented in this study, PV948712, PV948713, PV948715, PV948716, PV948717, PV948719, PV948721, and PV948724 presented the highest nucleotide similarities to the PQ043349 (HEV-3c; nt. ID 98.00%) observed in unpasteurized milk collected from the ovine species in the Abruzzo Region (Central Italy) (Ferri et al., 2024). The PV948714 showed an nt. ID of 99.32% to the ON908191.1 and ON908192.1 sequences that were detected in liver and diaphragm muscle tissues collected from hunted wild boars in the Marche region (Central Italy) (Ferri et al., 2022). Finally, the registered sequences belonging to this study, PV948718, PV948720, PV948722, PV948723, PV948725, PV948726, and PV948727, showed high nucleotide similarities (nt. ID 100.0%) to EF681109.1; this last-mentioned accession number refers to the HEV genotype 3 detected from swine stools farmed in Italy (Caprioli et al., 2007).

Concerning AstV, the obtained sequenced amplicons, ORF-2 gene fragments obtained from the electrophoresis assays, were analyzed by the BLASTN tool to identify the genus *Mamastrovirus* and were deposited and finally registered in the GenBank platform with the following identifiers: PV972762, PV972763, PV972764, PV972765, PV972766, PV972767, PV972768, and PV972769 (Table 3). All described sequences, belonging to this study, showed high nucleotide similarity (average nt. ID 99.0%) to the NC_002469, which was amplified for the first time in Europe from ovine feces in Norway as published by Jonassen et al. (2003). These were also very similar (nt. ID 96.0%) to the registered sequence OK107515 from caprine feces in China and to JK592482, a *Mamastrovirus* identified in Hungary by Reuter et al. (2012) in ovine feces.

The NoV virus, identified from all positive samples, belonged to genogroup I, and specific nucleotide sequences belonging to the *RdRp* genetic region were observed as products of the performed nested molecular screenings. Sequences were uploaded to the GenBank platform and released with the following accession numbers: PV948521, PV948700, PV948736, PV948859, PV948891, PV948902, PV948903, PV948904, PV948911, and PV948912, as schematically represented in Table 3. These ones showed high nucleotide similarities with NoV-GI (average nt. ID 99.64%) amplified from human patient stools discovered by Ramani et al. (2024) (GenBank accession number: PV242303) and (nt. 100.0%) to JF262163 amplified from human feces in Northern Italy by Di Bartolo et al. (2011).

## 4 Discussion

This study aimed to investigate the detection and circulation of viral RNA belonging to different emerging and re-emerging foodborne pathogens, including AstV, HAV, HEV, NoV-GI, NoV-GII, and RV, which represent public health concerns in caprine raw milk. Indeed, the observed findings represent the first report of viral co-harboring of RNA belonging to different zoonotic viruses in the same samples collected from dairy animals in Italy. The RNA viruses can be mainly eliminated from the environment by feces from the infected animal species. Virions can also gain different target cytotypes, such as mammary adenomas, resulting in potential excretion through the milk both in animal species and humans because of the estrogen effect during the lactation period. In general, it has been demonstrated that a hormone-related increased receptiveness in female hosts is higher than that observed in males because of the action of estrogen on different cytotypes (e.g., enterocytes, hepatocytes, etc.). Finally, in many productive systems, another contamination route for milk is represented by fecal contamination if sanitation and correct handling procedures are not sufficiently applied (Singh et al., 2019). The described pathways may represent risks for consumers (with special regard to the immunocompromised individuals) if milk and related products are consumed raw, without pasteurization processes (El-Senousy et al., 2020).

Based on this scientific rationale, this study performed an epidemiological investigation concerning the possible circulation of emerging and re-emerging viral foodborne pathogens in unpasteurized caprine milk. For this purpose, a total of 383 raw milk samples (one per animal) were collected from goats (*Capra aegagrus hircus*) farmed on six different sites (F1–F6), following the semi-extensive method, distributed in L’Aquila Province (Abruzzo Region, Italy). This zootechnical aspect has important implications for viral persistence in any ecological niche; indeed, the grazing sharing between domestic animal species and wild ones (acting as reservoirs) can represent a crucial variable that may have had possible repercussions on the epidemiological findings discovered in this study. The originality of this study is represented by the biomolecular investigation of the caprine raw milk, which has been designed to provide data on zoonotic viral circulation and on viral diversity.

Among the considered viruses, HEV RNA presented the highest prevalence value of 7.57% (29/383; 95% CI: 4.92–10.22%) compared with the other considered viral foodborne pathogens in this study. The obtained result is higher than the 2.80% discovered in caprine raw milk in the Czech Republic by Dziedzinska et al. (2020); however, the real-time RT-qPCR assays revealed similar loads ranging between 10^2^–10^3^ GE/mL. From a molecular quantitative perspective, El-Mokhtar et al. (2020) discovered similar HEV RNA amounts of 10^3^ GE/mL, but a low prevalence percentage of 0.7% after screening of a caprine population composed of 280 subjects. The detected values (GE/mL) have been demonstrated not to induce infection in immunocompetent human subjects with special regard to the HEV genotype 3 that has been identified in different animal species and humans in Europe. This genotype is well-known for sporadic hospitalizations (La Rosa et al., 2018).

On the contrary, the observed prevalence (7.57% or 29/383; 95% CI: 4.92–10.22%) observed in this study was lower than the 18.5% described by Demirci et al. (2019) in Turkey and 100.0% of tested samples in China by Long et al. (2017). These differences have a crucial common gap, which is represented by the limited number of tested animals per study; indeed, Long et al. (2017) screened four goats, and Demirci et al. (2019) included 12 animals in their studies. A further influencing variable is represented by the different farming systems; it is common in different Asiatic sites, the so-called mixed farms where different animal species (reservoirs and spillover species) share the same environments as humans (He et al., 2025). The HEV ultrastructural characteristics, which confer high resistance to different ecological conditions, have permitted the explanation of its persistence ability in a specific ecosystem (Ahmad et al., 2022). For this reason, HEV has been defined as an emerging and re-emerging foodborne viral pathogen (Lemon and Walker, 2019).

In Italy, HEV RNA was amplified only in 9.2% (9/119) of fecal samples collected from caprine farms in the Teramo province (Abruzzo Region) (Di Martino et al., 2016). However, from a comparative perspective, HEV RNA was amplified from the ovine species (farmed in three different Abruzzo provinces following the transhumance method) through the analyses of unpasteurized milk samples presenting the following prevalence value of 2.27% with amounts of 10^2^ GE/mL (Ferri et al., 2024). Therefore, the HEV RNA sequences discovered in this study in caprine raw milk samples represent the first Italian data from unprocessed animal-origin food. The scientific explanation, based on the inferences exposed by the previously mentioned studies about milk as a possible vector of viral particles, is based on the concept that the HEV RNA sequences were detected during peak lactation. This specific moment of lactation involves a crucial role for viral spreading within mammary tissues because of the increased vascularization, demonstrated to be a hormone-dependent response mediated by estrogen (Singh et al., 2019), with special regard to the viremia step (Rivero-Juarez et al., 2016).

Finally, the HEV-deposited and published sequences (Table 3) analyses permitted the identification of high nucleotide similarities with HEV RNA fragments discovered in reservoir animal species (hunted wild boars—GenBank: ON908191.1 and ON908192.1) and spillover wild or domestic ruminants (ovine—GenBank: PQ043349). This genetic evidence supports the scientific hypothesis that the environmental sharing for grazing between domestic and wild animal species can be considered a possible source for viral exposure and infection.

Concerning AstV RNA, it was observed in 6.00% (23/383; 95% CI:3.62–8.38%) of the screened milk samples; these data represent the first Italian detection in caprine raw milk. In the European scenario, the first genomic sequencing and registration were performed by Jonassen et al. (2003) (GenBank accession number: NC_002469) from ovine feces collected in Norway and from the same isolation source in Hungary (Reuter et al., 2012). It was also amplified by Kauer et al. (2019) from fecal samples from several small ruminant populations (goats, sheep, alpacas, and deer) farmed in Switzerland. Specifically, 0.48% of goats and 0.36% of sheep were positive. Both Jonassen et al. (2003) and Kauer et al. (2019) aimed to focus scientific attention on this emerging viral pathogen and its *silent* circulation among small and large ruminants, involving both domestic and wild animal species. Concerning the raw milk food matrix, AstV RNA was amplified from the 18.49% of screened bovines (population composed of 492 subjects) farmed in Iran, as described by Pakbin et al. (2022). From the scientific literature, it emerges that AstV RNA sequences have been marginally amplified from both fecal and raw milk samples obtained from dairy animal species; however, their harboring and co-harboring in milk samples could contribute as a *stimulus* for further investigation to identify and understand possible triggers in the interactions between viral pathogens and hosts.

Recently discussed considerations focus on the NoV-GI RNA sequences detected from the 5.74% (22/383; 95% CI: 3.41–8.07%) of the 383 caprine raw milk samples. Although it has been largely amplified from bivalve lamellibranchs (Blanda et al., 2024), the prevalence reported in this study represents the first Italian data of NoV-GI detection from unpasteurized caprine milk specimens. The comparisons between this study and the literature highlighted that the obtained prevalence was higher than the 2.43% reported by Pakbin et al. (2022) from raw milk specimens collected from cows farmed in Iran. In accordance with the previous similar study, NoV-GI RNA detection indicates fecal contamination, although the low amounts of 10^1^ GE/mL detected in this study imply the necessity of improving hygienic procedures applied before and after the milk collection (Boros et al., 2023).

When comparing the virological results to the hygienic bacteriological findings, the tested raw milk samples, collected from the six (F1–F6) above-mentioned farms (in accordance with their analytical plans), showed the total bacterial counts (TBC) and total coliform (TCC) counts (expressed as averages): 4.90 log CFU/mL and 3.57 log CFU/mL. When comparing these profiles to similar studies on caprine raw milk, the observed bacterial loads were lower than those of Aliyo et al. (2022), with TBC 7.75 log CFU/mL and TCC 6.69 log CFU/mL, but consistent with TBC 4.57 log CFU/mL and TCC 3.69 log CFU/mL reported by Metz et al. (2020). Both studies suggested the consistent importance of high, standardized hygienic procedures as strategic preventive tools. Although the detected bacterial loads highlight appreciable hygienic levels, the virological results, in contrast, represent another microbiological scenario. Indeed, the detected viral RNA diversity, observed in this study, does not represent tangible evidence of viral infectivity, but its harboring wants to highlight a *silent* circulation of genetic sequences and the exposure of food-producing animals to infections caused by many viral pathogens. However, it is important to note that the pasteurization and/or boiling processes have significant virucidal effects (Huang et al., 2016; Santos-Silva et al., 2022), representing the majority of effective sanitation methods to avoid foodborne infections for final consumers.

The detected co-detection profiles suggest co-circulation of multiple RNA fragments, belonging to AstV, HEV, and NoV-GI, supporting the scientific hypothesis about the complexity of multiviral detections in organic matrices (including milk), which can facilitate host susceptibility to different viral infections (Boros et al., 2023). The data showed that 15.40% (59/383; 95% CI: 11.79–19.01%) of unpasteurized milk samples were positive for RNA sequences of one of the five screened viral pathogens. Among positive milk samples, multiple co-harboring profiles were observed; indeed, 22.03% (13/59; 95% CI: 11.46–32.60%) of them were 2-CoH (84.61% or 11/13; 95% CI: 65.00–100.00%) and 3-CoH (15.38% or 2/13; 95% CI: 0.02–30.74%). AstV RNA sequences were detected in all CoH-positive samples. This microbiological behavior was first described by Boros et al. (2023), who observed that the enteric virome and viral diversity can characterize and affect small ruminants (with special regard to the domestic goat) and humans, highlighting the possibility of combined infections and the consequent effects on the host immune responses.

From a comparative perspective, the 2-CoH and/or 3-CoH viral profiles described in this study have not been observed in the caprine species but have been reported in bovines. Indeed, Pakbin et al. (2022) conducted an investigation on the prevalence of several foodborne and zoonotic viruses (AstV, HAV, NoV-GI, NoV-GII, RV, bovine leukemia virus, and the tickborne encephalitis virus) in unpasteurized milk samples collected from 492 cows. They observed different combinations of 2-CoH, 3-CoH, or multiple profiles; specifically, the AstV RNA sequences were mainly found in the 2-CoH and 3-CoH forms, representing 26.0% of screened positive specimens. This percentage value is in line with that of 22.03% detected in this study. Comparing Pakbin et al. (2022) and this study, the data highlight agreement about the co-detection of AstV RNA amplifications with other viruses such as NoV-GI, HAV, and HEV. In this study, the AstV-NoV-GI co-detection was observed in 6.77% (4/59; 95% CI: 0.36–13.18%) of positive samples, and the observed prevalence value was lower than the 15.24% described by Pakbin et al. (2022). Furthermore, AstV and HEV RNA sequences were both detected in 11.86% (7/59; 95% CI: 3.61–20.11%) of positive samples in this study and, in contrast, were combined with HAV RNA in 5.28%, as reported by Pakbin et al. (2022). The scientific explanation of dual or multiple viral RNA harboring has been suggested to correlate (Demirci et al., 2019; Dziedzinska et al., 2020; El-Senousy et al., 2020; Ferri et al., 2024) with poor hand hygiene of infected handlers and contaminated tank and tube surfaces. In addition, the environmental persistence of the considered viruses is facilitated by their ultrastructural characteristics, which confer high resistance, preserving their infectivity; indeed, Pexara and Govaris (2020) demonstrated this concept in fresh dairy products, milk production plants, and wastewaters. This highlighted the lack and limitations of safe effluent management at treatment systems (used in different production settings, e.g., food industries, urban wastewater, etc.) for viral pathogens that, on the contrary, demonstrated the ability to reduce bacterial loads (Bleotu et al., 2024).

Following these affirmations about the co-detections, the following paragraphs will discuss the results observed for each viral pathogen.

Furthermore, the real complexity emerges in the milk specimens and their respective virome profiles, which depend on many variables, e.g., environmental sharing among susceptible animal reservoirs and spillover, zootechnical farming systems, viral ultrastructural characteristics, wastewater management, environmental viral persistence, etc. These aspects contribute to the viral detections (in both reservoir and spillover animal species and humans), which must be studied in depth following intersectoral links to support epidemiological assessments with consequential repercussions on the environments and animal-origin production systems. This study has some limitations that should be considered. First, the data collection was based mainly on the molecular methods without an in-depth analysis of clinical aspects, and finally, the lack of long-term follow-up does not allow for the evaluation of the infection trends over time. Further studies involving larger and more diverse populations will be useful to confirm and further explore these findings.

## 5 Conclusion

This is the first report from Italy showing multiple viral circulations in caprine raw milk. These results show viral diversity in an unprocessed food matrix that silently circulates among dairy-receptive hosts, confirming the need to pay greater attention to possible cross-species transmission, including to humans. Based on their oro-fecal routes of host infection, food safety implications are involved in order to ensure consumer health. Although the screened specimens are raw food matrices, the pasteurization process, depending on the initial viral loads, could be effective as a virucidal process; boiling remains the safest method. These One Health sanitary implications also highlight the importance of improving and better applying mammary sanitation procedures, biosecurity levels at the farm level, and biomolecular surveillance plans in order to contribute to reducing viral circulation among different ecosystems. These concerns should be supported by the European legislator by including more viral pathogens within both food safety criteria and food hygiene processes addressed in the EU Regulation No. 2073/2005. Although this study has been performed in a specific Italian region, this evidence contributes to providing data and drawing attention to this viral underexplored world, which can be considered as underestimated if compared to bacterial pathogens.

## Data Availability

The data presented in the study are included in the article, further inquiries can be directed to the corresponding author.

## Footnotes

1 https://blast.ncbi.nlm.nih.gov/Blast.cgi?PROGRAM=blastn&BLAST_SPEC=GeoBlast&PAGE_TYPE=BlastSearch (Accessed May 03 and 12, 2025).

2
https://www.ncbi.nlm.nih.gov/genbank/

## References

[ref1] Ahmad T. Jin H. Dhama K. Yatoo M. I. Tiwari R. Bilal M. . (2022). Hepatitis E virus in pigs and the environment: an updated review of public health concerns. Narra J 2:e78. doi: 10.52225/narra.v2i2.78, 38449702 PMC10914032

[ref2] Aliyo A. Seyoum A. Teklemariam Z. (2022). Bacteriological quality and antimicrobial susceptibility patterns among raw milk producers and vendors in Gomole District, Borena zone, southern Ethiopia. Infect. Drug Resist. 15, 2589–2602. doi: 10.2147/IDR.S364578, 35619734 PMC9128746

[ref3] Ben Salem A. N. Chupin Sergei A. Bjadovskaya Olga P. Andreeva Olga G. Mahjoub A. Prokhvatilova L. B. (2010). Multiplex nested RT-PCR for the detection of porcine enteric viruses. J. Virol. Methods 165, 283–293. doi: 10.1016/j.jviromet.2010.02.010, 20170679 PMC7112813

[ref4] Blanda V. Giacchino I. Vaglica V. Milioto V. Migliore S. Di Bella S. . (2024). Foodborne pathogens across different food matrices in Sicily (southern Italy). Pathogens (Basel, Switzerland) 13:998. doi: 10.3390/pathogens13110998, 39599551 PMC11597087

[ref5] Bleotu C. Matei L. Dragu L. D. Necula L. G. Pitica I. M. Chivu-Economescu M. . (2024). Viruses in wastewater-a concern for public health and the environment. Microorganisms 12:1430. doi: 10.3390/microorganisms12071430, 39065197 PMC11278728

[ref6] Boros Á. Pankovics P. László Z. Urbán P. Herczeg R. Gáspár G. . (2023). The genomic and epidemiological investigations of enteric viruses of domestic caprine (*Capra hircus*) revealed the presence of multiple novel viruses related to known strains of humans and ruminant livestock species. Microbiol. Spectrum 11:e0253323. doi: 10.1128/spectrum.02533-23, 37823638 PMC10714811

[ref7] Bosch A. Guix S. Krishna N. K. (2019). ICTV virus taxonomy profile: Astroviridae. J. Gen. Virol. 100, 1598–1599.

[ref8] Caprioli A. Martelli F. Ostanello F. Di Bartolo I. Ruggeri F. M. Del Chiaro L. . (2007). Detection of hepatitis E virus in Italian pig herds. Vet. Rec. 161, 422–423. doi: 10.1136/vr.161.12.422, 17890772

[ref9] Demirci M. Yiğin A. Ünlü Ö. Kılıç Altun S. (2019). Farklı hayvanlardan elde edilen çiğ sütlerde HEV RNA miktarının ve genotiplerinin tespiti [detection of HEV RNA amounts and genotypes in raw milks obtained from different animals]. Mikrobiyoloji bul 53, 43–52. doi: 10.5578/mb.67468, 30683038

[ref10] Di Bartolo I. Monini M. Losio M. N. Pavoni E. Lavazza A. Ruggeri F. M. (2011). Molecular characterization of noroviruses and rotaviruses involved in a large outbreak of gastroenteritis in northern Italy. Appl. Environ. Microbiol. 77, 5545–5548. doi: 10.1128/AEM.00278-11, 21666024 PMC3147480

[ref11] Di Martino B. Di Profio F. Melegari I. Sarchese V. Robetto S. Marsilio F. . (2016). Detection of hepatitis E virus (HEV) in goats. Virus Res. 225, 69–72. doi: 10.1016/j.virusres.2016.09.008, 27647265

[ref12] Dziedzinska R. Krzyzankova M. Bena M. Vasickova P. (2020). Evidence of hepatitis E virus in goat and sheep Milk. Viruses 12:1429. doi: 10.3390/v12121429, 33322702 PMC7763044

[ref13] El-Mokhtar M. A. Elkhawaga A. A. Sayed I. M. (2020). Assessment of hepatitis E virus (HEV) in the edible goat products pointed out a risk for human infection in upper Egypt. Int. J. Food Microbiol. 330:108784. doi: 10.1016/j.ijfoodmicro.2020.108784, 32659521

[ref14] El-Senousy W. M. Shalaby M. Deeb A. M. M. Alhawary I. I. (2020). Thermal inactivation of hepatitis a virus, noroviruses, and simian rotavirus in cows' milk. Food Environ. Virol. 12, 310–320. doi: 10.1007/s12560-020-09443-z, 32930960

[ref15] Ferri G. Lauteri C. Festino A. R. Piccinini A. Olivastri A. Vergara A. (2022). Hepatitis E virus detection in hunted wild boar liver and muscle tissues in Central Italy. Microorganisms 10:1628. doi: 10.3390/microorganisms10081628, 36014046 PMC9414245

[ref16] Ferri G. Pennisi L. Malatesta F. Vergara A. (2024). First detection of hepatitis E virus RNA in ovine raw Milk from herds in Central Italy. Foods (Basel, Switzerland) 13:3218. doi: 10.3390/foods13203218, 39456280 PMC11507303

[ref17] Fusco V. Chieffi D. Fanelli F. Logrieco A. F. Cho G. S. Kabisch J. . (2020). Microbial quality and safety of milk and milk products in the 21st century. Compr. Rev. Food. Sci. Food Saf. 19, 2013–2049. doi: 10.1111/1541-4337.12568, 33337106

[ref18] He Z. Liu D. Liu B. Zhang P. Wang X. Wang G. . (2025). Prevalence of hepatitis E virus in swine in China: a systematic review with meta-analysis (2004-2023). Front. Vet. Sci. 11:1472658. doi: 10.3389/fvets.2024.1472658, 40084118 PMC11905393

[ref19] Huang F. F. Haqshenas G. Guenette D. K. Halbur P. G. Schommer S. K. Pierson F. W. . (2002). Detection by reverse transcription-PCR and genetic characterization of field isolates of swine hepatitis E virus from pigs in different geographic regions of the United States. J. Clin. Microbiol. 40, 1326–1332. doi: 10.1128/JCM.40.4.1326-1332.2002, 11923352 PMC140370

[ref20] Huang F. Li Y. Yu W. Jing S. Wang J. Long F. . (2016). Excretion of infectious hepatitis E virus into milk in cows imposes high risks of zoonosis. Hepatol 64, 350–359. doi: 10.1002/hep.28668, 27286751

[ref21] Johne R. Plenge-Bönig A. Hess M. Ulrich R. G. Reetz J. Schielke A. (2010). Detection of a novel hepatitis E-like virus in faeces of wild rats using a nested broad-spectrum RT-PCR. J. Gen. Virol. 91, 750–758. doi: 10.1099/vir.0.016584-0, 19889929

[ref22] Jonassen C. M. Jonassen T. T. Ø. Sveen T. M. Grinde B. (2003). Complete genomic sequences of astroviruses from sheep and Turkey: comparison with related viruses. Virus Res. 91, 195–201. doi: 10.1016/s0168-1702(02)00269-1, 12573498

[ref23] Karesh W. B. Dobson A. Lloyd-Smith J. O. Lubroth J. Dixon M. A. Bennett M. . (2012). Ecology of zoonoses: natural and unnatural histories. Lancet 380, 1936–1945. doi: 10.1016/S0140-6736(12)61678-X, 23200502 PMC7138068

[ref24] Kauer R. V. Koch M. C. Hierweger M. M. Werder S. Boujon C. L. Seuberlich T. (2019). Discovery of novel astrovirus genotype species in small ruminants. PeerJ. 7:e7338. doi: 10.7717/peerj.7338, 31396439 PMC6679648

[ref26] Kralik P. Ricchi M. (2017). A basic guide to real time PCR in microbial diagnostics: definitions, parameters, and everything. Front. Microbiol. 8:108. doi: 10.3389/fmicb.2017.00108, 28210243 PMC5288344

[ref27] Kumar S. Stecher G. Li M. Knyaz C. Tamura K. (2018). MEGA X: molecular evolutionary genetics analysis across computing platforms. Mol. Biol. Evol. 35, 1547–1549. doi: 10.1093/molbev/msy096, 29722887 PMC5967553

[ref28] La Rosa G. Proroga Y. T. R. De Medici D. Capuano F. Iaconelli M. Della Libera S. . (2018). First detection of hepatitis E virus in shellfish and in seawater from production areas in southern Italy. Food Environ. Virol. 10, 127–131. doi: 10.1007/s12560-017-9319-z, 28956272

[ref29] Lemon S. M. Walker C. M. (2019). Hepatitis a virus and hepatitis E virus: emerging and re-emerging enterically transmitted hepatitis viruses. Cold Spring Harb. Perspect. Med. 9:a031823. doi: 10.1101/cshperspect.a031823, 29735577 PMC6531368

[ref30] Lew J. F. Petric M. Kapikian A. Z. Jiang X. Estes M. K. Green K. Y. (1994). Identification of minireovirus as a Norwalk-like virus in pediatric patients with gastroenteritis. J. Virol. 68, 3391–3396. doi: 10.1128/JVI.68.5.3391-3396.1994, 8151799 PMC236832

[ref31] Long F. Yu W. Yang C. Wang J. Li Y. Li Y. . (2017). High prevalence of hepatitis E virus infection in goats. J. Med. Virol. 89, 1981–1987. doi: 10.1002/jmv.24843, 28464334

[ref32] Matthijnssens J. Attoui H. Bányai K. Brussaard C. P. D. Danthi P. Del Vas M. . (2022). ICTV Virus Taxonomy Profile: *Sedoreoviridae* 2022. J. Gen. Virol. 103:001782. doi: 10.1099/jgv.0.001782, 36215107 PMC12643109

[ref33] Metz M. Sheehan J. Feng P. C. H. (2020). Use of indicator bacteria for monitoring sanitary quality of raw milk cheeses - a literature review. Food Microbiol. 85:103283. doi: 10.1016/j.fm.2019.103283 31500718

[ref34] Mikel P. Vasickova P. Tesarik R. Malenovska H. Kulich P. Vesely T. . (2016). Preparation of MS2 phage-like particles and their use as potential process control viruses for detection and quantification of enteric RNA viruses in different matrices. Front. Microbiol. 7:1911. doi: 10.3389/fmicb.2016.01911, 28133456 PMC5234545

[ref35] Noel J. S. Lee T. W. Kurtz J. B. Glass R. I. Monroe S. S. (1995). Typing of human astroviruses from clinical isolates by enzyme immunoassay and nucleotide sequencing. J. Clin. Microbiol. 33, 797–801. doi: 10.1128/jcm.33.4.797-801.1995, 7790440 PMC228043

[ref36] Ozogul F. Rathod N. Köse S. Alak G. Kızılyıldırım S. Bilgin Ş. . (2025). Biochemical and microbial food safety hazards in seafood: a Mediterranean perspective (part 2). Adv. Food Nutr. Res. 114, 209–271. doi: 10.1016/bs.afnr.2024.09.003, 40155085

[ref37] Pakbin B. Rossen J. W. A. Brück W. M. Montazeri N. Allahyari S. Dibazar S. P. . (2022). Prevalence of foodborne and zoonotic viral pathogens in raw cow milk samples. FEMS Microbiol. Lett. 369:fnac108. doi: 10.1093/femsle/fnac108, 36352488

[ref38] Pexara A. Govaris A. (2020). Foodborne viruses and innovative non-thermal food-processing technologies. Foods (Basel, Switzerland) 9:1520. doi: 10.3390/foods9111520, 33113926 PMC7690672

[ref39] Purdy M. A. Drexler J. F. Meng X. J. Norder H. Okamoto H. Van der Poel W. H. M. . (2022). ICTV virus taxonomy profile: Hepeviridae 2022. J. Gen. Virol. 103:001778. doi: 10.1099/jgv.0.001778, 36170152 PMC12642825

[ref40] Purdy M. A. Harrison T. J. Jameel S. Meng X. J. Okamoto H. Van der Poel W. H. M. . (2017). ICTV virus taxonomy profile: *Hepeviridae*. J. Gen. Virol. 98, 2645–2646. doi: 10.1099/jgv.0.000940 29022866 PMC5718254

[ref41] Ramani S. Javornik Cregeen S. J. Surathu A. Neill F. H. Muzny D. M. Doddapaneni H. . (2024). Intra- and inter-host evolution of human norovirus in healthy adults. bioRxiv. doi: 10.1101/2023.05.30.542907

[ref42] Regional Abruzzo Hunting Report and ISPRA (2023). Report Attività Faunistico-Venatoria. Available online: at: https://www.regione.abruzzo.it/system/files/caccia-pesca/notizie/170701/allegato-ii.pdf (Accessed January 12, 2024).

[ref43] Reuter G. Pankovics P. Delwart E. Boros Á. (2012). Identification of a novel astrovirus in domestic sheep in Hungary. Arch. Virol. 157, 323–327. doi: 10.1007/s00705-011-1151-4, 22033597 PMC3518301

[ref44] Rivero-Juarez A. Frias M. Rodriguez-Cano D. Cuenca-López F. Rivero A. (2016). Isolation of hepatitis E virus from breast Milk during acute infection. Clin. Infect. Dis. 62:1464. doi: 10.1093/cid/ciw186 27025819

[ref45] Saitou N. Nei M. (1987). The neighbor-joining method: a new method for reconstructing phylogenetic trees. Mol. Biol. Evol. 4, 406–425. doi: 10.1093/oxfordjournals.molbev.a040454, 3447015

[ref46] Santos-Silva S. Gonçalves H. M. R. Rivero-Juarez A. Van der Poel W. H. M. Nascimento M. S. J. Mesquita J. R. (2022). Detection of hepatitis E virus in milk: current evidence for viral excretion in a wide range of mammalian hosts. Transbound. Emerg. Dis. 69, 3173–3180. doi: 10.1111/tbed.14683, 35989468

[ref47] Singh S. Daga M. K. Kumar A. Husain S. A. Kar P. (2019). Role of oestrogen and its receptors in HEV-associated feto-maternal outcomes. Liver Int. 39, 633–639. doi: 10.1111/liv.13928, 29979823

[ref48] Taffon S. Bidini G. Vichi F. Corti G. Genovese D. Kondili L. A. . (2011). A unique HAV strain circulated in patients with acute HAV infection with different risk exposures in Tuscany, Italy. J. Clin. Virol. 50, 142–147. doi: 10.1016/j.jcv.2010.10.011, 21094625

[ref49] Vinjé J. Estes M. K. Esteves P. Green K. Y. Katayama K. Knowles N. J. . (2019). ICTV virus taxonomy profile: *Caliciviridae*. J. Gen. Virol. 100, 1469–1470. doi: 10.1099/jgv.0.001332 31573467 PMC7011698

[ref51] Zell R. Delwart E. Gorbalenya A. E. Hovi T. King A. M. Q. Knowles N. J. . (2017). ICTV virus taxonomy profile: Picornaviridae. J. Gen. Virol. 98, 2421–2422. doi: 10.1099/jgv.0.000911 28884666 PMC5725991

